# Routine immunization in Pakistan: comparison of multiple data sources and identification of factors associated with vaccination

**DOI:** 10.1093/inthealth/ihx067

**Published:** 2018-02-08

**Authors:** Hafsa Imran, Dania Raja, Nicholas C Grassly, M Zubair Wadood, Rana M Safdar, Kathleen M O’Reilly

**Affiliations:** 1 MRC Centre for Outbreak Analysis and Modelling, Department of Infectious Disease Epidemiology, St Mary’s Campus, Imperial College London, London, UK; 2 World Health Organization, Islamabad, Pakistan; 3 National Emergency Operation Centre, Ministry of National Health Services, Regulations and Coordination, Islamabad, Pakistan

**Keywords:** Education, Measles, Poliomyelitis, Routine immunization, Vaccination

## Abstract

**Background:**

Within Pakistan, estimates of vaccination coverage with the pentavalent vaccine, oral polio vaccine (OPV) and measles vaccine (MV) in 2011 were reported to be 74%, 75% and 53%, respectively. These national estimates may mask regional variation. The reasons for this variation have not been explored.

**Methods:**

Data from the Multiple Indicator Cluster Surveys (MICS) for Balochistan and Punjab (2010–2011) are analysed to examine factors associated with receiving three or more doses of the pentavalent vaccine and one or more MVs using regression modelling. Pentavalent and OPV estimates from the MICS were compared to vaccine dose histories from surveillance for acute flaccid paralysis (AFP; poliomyelitis) to ascertain agreement.

**Results:**

Adjusted coverage of children 12–23 months of age were estimated to be 16.0%, 75.5% and 34.2% in Balochistan and 58.0%, 87.7% and 72.6% in Punjab for the pentavalent vaccine, OPV and MV, respectively. Maternal education, healthcare utilization and wealth were associated with receiving the pentavalent vaccine and the MV. There was a strong correlation of district estimates of vaccination coverage between AFP and MICS data, but AFP estimates of pentavalent coverage in Punjab were biased toward higher values.

**Conclusions:**

National estimates mask variation and estimates from individual surveys should be considered alongside other estimates. The development of strategies targeted towards poorly educated parents within low-wealth quintiles that may not typically access healthcare could improve vaccination rates.

## Introduction

Vaccines are among the greatest success stories in public health. Since 2005 the average global child mortality rate has fallen by 3.6% per year^1^ and Wang et al.^1^ estimate that the impact of ‘secular trends’, which include technological developments such as vaccines, had the largest impact on reductions in child mortality rates. However, within this comprehensive analysis, nine countries were highlighted as having child mortality rates that were decreasing at a lower-than-expected rate. Pakistan is one such country, where between 2000 and 2013 an average 1.8% reduction in childhood mortality was reported, and in 2013 there was an average 75.8 deaths per 1000 live births. As well as reporting a slow decline in childhood mortality, Pakistan is one of the three remaining countries that has yet to interrupt poliovirus transmission. Since 2000 the annual number of cases has ranged from 20 to 306, with substantial resources put into vaccination and surveillance to achieve eradication. Measles remains endemic within the country, and although surveillance is not as comprehensive as for poliomyelitis, outbreaks are frequently reported. In 2013, 14 000 cases of measles were reported, resulting in 306 deaths in Pakistan.^2^ Identifying regional variations in vaccination coverage and associations with demographic factors may assist with the development of strategies to improve coverage.

To combat vaccine-preventable diseases, the Expanded Programme on Immunization (EPI) was launched in Pakistan in 1978.^3^ A widely recognized marker of a good EPI programme is the percentage of children 12–24 months of age who have received at least three doses of the pentavalent vaccine (or equivalent, which protects against *Haemophilus influenza* B, diphtheria, pertussis, tetanus and hepatitis B). In Pakistan, children receive the pentavalent vaccine at 6, 10 and 14 weeks, coinciding with routine administration of the oral polio vaccine (OPV). Two doses of the measles vaccine (MV), administered by 9 and 12 months of age, were introduced into the EPI schedule in the early 1980s, and several other immunogens have been recently introduced.^4^ Within Pakistan, estimates of vaccination coverage with the pentavalent vaccine, OPV and MV in 2011 were reported to be 74%, 75% and 53%, respectively. Vaccines that are part of the EPI are free of charge, but their use may be limited by access to healthcare and a caregiver’s willingness to seek vaccination for his/her children.^5^

Many studies suggest that increased parental education (secondary level or higher) and the attitude of mothers regarding vaccination are the main determinants of improved coverage. There are likely to be many reasons for this strong link, including promotion of health literacy and enabling women to be part of decision making within the family.^6–8^ As Pakistan is an agricultural-based economy, with 62% of the population residing in rural villages and limited adult education, there may be a urban–rural divide in vaccination rates. An analysis of Demographic Health Survey data in Pakistan from 2006 identified these factors, in addition to the father’s occupation (professional vs manual/unemployed), access to information and hospital delivery of children (vs home delivery), to be positively associated with increased routine immunization.^9^ Here we utilize Multiple Indicator Cluster Survey (MICS) data that were collected between 2010 and 2011 to assess coverage and factors associated with improved coverage.

To monitor progress made in improving vaccination coverage within countries, cross-sectional surveys are used to measure coverage. Approximately 20 surveys per year are carried out globally, either as a Demographic Health Survey (DHS)^10^ or MICS,^11^ and these household surveys collect information on a variety of health-related information. Cross-sectional surveys such as these may be prone to biases in selection of households within areas that are easy to access and may overinflate estimates of coverage in the general population.^13^ Additionally, the use of caregiver’s recall to estimate coverage may be prone to an upward bias due to difficulties in recalling receipt of specific vaccines and interviewer pressure to record receipt of vaccines. It is therefore important to compare coverage estimates from household surveys with other available sources of information. In addition to identifying factors associated with routine vaccination, we compared reported coverage of the pentavalent vaccine and OPV to vaccine dosage history from polio surveillance, which is not prone to the same geographical selection bias. We hypothesize that the use of vaccination coverage data from surveillance for acute flaccid paralysis (AFP) may be less prone to selection bias, as the health-seeking behaviour of caregivers of children with AFP should be consistent across all settings.

## Materials and methods

Pakistan consists of four provinces (Balochistan, Punjab, Sindh and Khyber Pakhtunkhwa), one federal capital territory (Islamabad), a group of federally administered tribal areas and two autonomous and disputed territories. The surveys described here were carried out in two of the four provinces.

### Questionnaire and sample size

The MICS is a household survey program developed by UNICEF. In Pakistan, surveys were carried out in Balochistan and Punjab in 2010 and 2011, respectively.^11^ In both cases the study design was a two-stage stratified cluster sampling approach where sample sizes and the methodological approach were designed to obtain reliable estimates of indicators for rural and urban settings within regions for Balochistan and for tehsils (smaller divisions than districts) in Punjab. The population size of each province estimated by the most recent (1988) census was 73.6 million in Punjab and 6.5 million in Balochistan.^12^ The sample weights are provided as part of the publically available information from the survey and were based on population estimates from the census. At the time of writing (March 2017), only surveys from Balochistan and Punjab were available for analysis.

The questions within the MICS are divided into three categories: household, women and children (<5 y old). There are more than 100 indicators used in the survey, and we use a hypotheses-driven approach to select variables to examine using regression modelling. The MICS data consist of 5736 observations from Balochistan and 74 126 from Punjab. The analysis was restricted to observations of children 0–2 y of age and those with usable information on pentavalent, OPV and MV history.

### Surveillance for poliomyelitis cases

Surveillance for poliomyelitis is based on reporting of AFP by healthcare providers.^14^ AFP surveillance is intensive, especially in endemic countries such as Pakistan, where the non-polio AFP detection rate in 2010–2012 was >8 cases per 100 000 children <15 y of age.^14^ Two stool samples are collected within 14 d of the onset of paralysis and >24 h apart, and are tested for the presence of poliovirus. At the time of AFP investigation, an interview with the parents/caregivers of the child is conducted where information on the reported number of OPV doses received through routine immunization and supplementary immunization activities (SIAs) is sought. Countries within the Eastern Mediterranean WHO Region report this vaccine history from routine immunization and SIAs separately, enabling a comparison of coverage with that reported in household surveys. Cases in which two adequate stool samples are found to be negative for both wild-type and vaccine-related polioviruses are defined as non-polio AFP. Non-polio AFP cases from children 0–2 y of age with onset of symptoms between 1 January 2010 and 31 December 2011 within Balochistan and Punjab were selected for analysis.

### Data analysis

Estimates of the adjusted percentage of children <2 years of age that had received at least one MV dose and at least three pentavalent and OPV doses were reported by province. Additionally, variation in coverage by district groups (Balochistan) and district (Punjab) was also reported and these district estimates form the basis of comparisons with the AFP data.

Associations of routine vaccination with potential explanatory variables were examined using regression modelling, accounting for the sample weights and non-response.^15^ The Balochistan and Punjab surveys were combined and the observation weights were adjusted to account for combining the surveys. Factors associated with receiving at least three pentavalent doses and at least one MV dose were examined separately using a regression model with logistic link function. Explanatory variables included within Bugvi et al.^9^ were tested in the current dataset and cover factors relating to parental characteristics, place of residence, gender, birth order, place of delivery, use of antenatal care and wealth index. Variables were screened using a univariate model for each explanatory variable where age (in years) was forced into the model. The unconditional model implicitly includes age as a regression coefficient. Variables significant (p<0.05) at the univariate level were combined into a multivariate model using a backward stepwise approach. With weighted observations, model building was carried using a series of F tests on each potential explanatory variable and candidate models were compared using area under the curve (AUC) diagnostics. The model consists of estimating the probability that a randomly selected child *i* is immunized with three or more doses of the pentavalent vaccine or one dose of the MV, $y_{i}\sim \mathrm{binomial}(p_{i},1)$. The regression modelling tests whether the probability of success is associated with individual-level variables: i.e., $p_{i}\;=\;\beta_{0}\;+\;\beta_{1}x_{i,1}w_{i}+\ldots +\beta_{k}x_{i,k}w_{i}\;+\;\varepsilon_{i}$. The parameters of the model consist of the intercept (*β*_0_), the fixed effects for each of the *k* variables in the model (*β*_*k*_), the observations from child *i* (*x*_*i,k*_), the weight of child *i* sampled in the surveys (*w*_*i*_) and random error *ε*_*i*_.

The percentage of children within each district age group with at least three OPV doses from routine immunization were reported from the non-polio AFP data. This estimate was compared with the pentavalent coverage within the MICS data for both Balochistan and Punjab, as routine pentavalent vaccine and routine OPV are received during the same visit to the health centre. The data sources were compared by examining the correlation between values using Pearson’s product moment correlation and the differences between values were explored to examine the percentage error between observations. The average total polio doses reported for each district age group within each dataset were also compared. Preliminary analysis indicated that within the Punjab MICS, seven polio doses may have been used as an upper limit, as 48% of observations reported this value. While the intention was to compare total OPV doses reported within the AFP and MICS data, only data from Balochistan were compared. The AFP data do not include information on whether the vaccination card was available and thus all AFP data are compared to all MICS data (based on records with and without vaccination cards).

The analysis was carried out in R (version 3.3.2; R Project for Statistical Computing, Vienna, Austria) and Stata (using the svy tools; StataCorp, College Station, TX, USA). The code used for the analysis is available online (https://github.com/kath-o-reilly/pakistan-routinevacc).

## Results

A total of 25 302 observations (32% of the total) from the MICS within Balochistan and Punjab were included in the analysis. The large reduction in observations was due to insufficient information on vaccination history being available (66% of all observations, where most observations were recorded as blank entries) or age not being recorded (the remaining 1%).

### Estimates of routine immunization coverage

**Figure 1.**
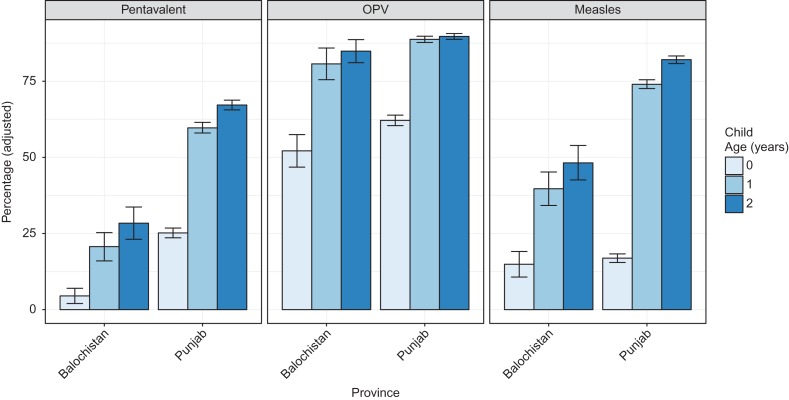
Adjusted percentage reporting at least three doses of the pentavalent vaccine, the OPV and at least one dose of the MV by age and province in Pakistan. The reported percentage accounts for the sampling frame of the surveys, bars indicate the 95% CIs.

**Figure 2.**
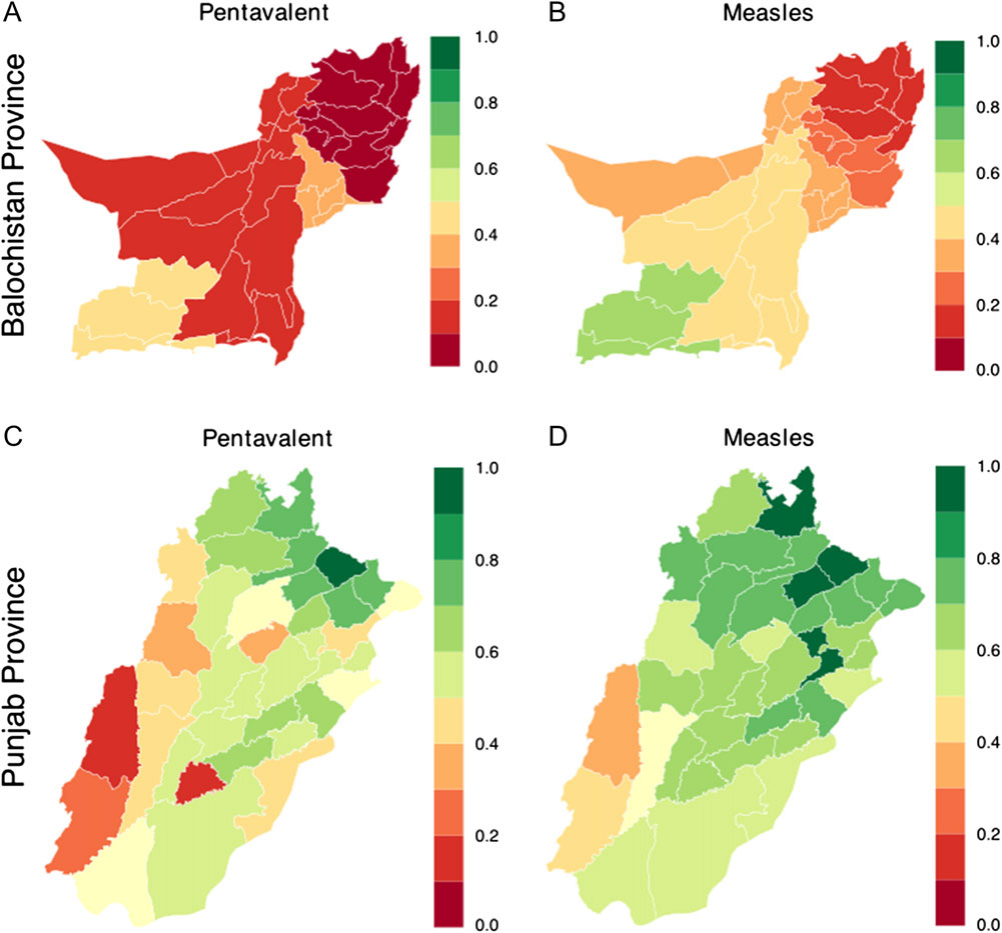
Region (Balochistan) and district (Punjab) estimates of coverage with at least three doses of the pentavalent vaccine and at least one dose of the MV in children 12–23 months of age. Estimates are from the MICS.

For each vaccine, we report by province and age the adjusted percentage of children who received at least one dose as reported in the MIC surveys (Figure 1). For all vaccines, children within Punjab had higher coverage than those in Balochistan. Children 12–23 months of age in Balochistan had a 20.7%, 80.7% and 39.7% chance of receiving three or more pentavalent doses, three or more OPV doses and at least one MV dose, respectively. Within Punjab, children 12–23 months of age had a 59.7%, 88.8% and 74.0% chance of receiving the same vaccines, respectively. The Balochistan survey was designed to estimate coverage for regions (consisting of several connected districts) within the province, where the regional coverage varied from 0.0% (Sibi region) to 45.7% (Mekran region) for the pentavalent vaccine and from 16.7% (Zhob) to 78.3% (Mekran region) for the MV (Figure 2A and B). The Punjab survey was sufficiently powered to estimate coverage within districts, and coverage varied from 17.2% (Lodhran) to 92.0% (Gujrat) for the pentavalent vaccine and from 31.4% (Dera Ghazi Khan) to 95.0% (Gujrat) for the MV (Figure 2C and D).

### Factors associated with routine immunization

In the final multivariate model for pentavalent vaccination, possession of a vaccination card, mothers educated beyond primary level, access to a television and wealth index were independently associated with an increased probability of receiving the full pentavalent schedule (Table 1). Additionally, access to a radio was associated with a reduced probability of receiving the full pentavalent schedule. The full model had an AUC estimate of 0.752, compared with the unconditional model, which had a value of 0.660. Factors independently associated with MV coverage included possession of a vaccination card, use of antenatal care, the child being born within a government hospital, access to a TV and wealth index. Inclusion of these variables in a multivariate model increased the AUC from 0.772 to 0.850. For some variables within the final multivariate model the proportion of missing observations was high, including the place of the child’s birth (28.7% of observations) and the education of the mother (53.6% of observations).

**Table 1.** Factors associated with pentavalent and measles immunization in Balochistan and Punjab

Variables	Factors	Observations (% unweighted) [% weighted]	At least three doses of the pentavalent vaccine		At least one dose of the MV	
			p-Value	OR (95% CI)	p-Value	OR (95% CI)
Vaccination card	No	15 640 (66.2) [67.3]		Baseline		Baseline
	Yes, not seen	7953 (33.7) [32.6]	<0.001	2.54 (2.1 to 3.08)	<0.001	3.11 (2.55 to 3.78)
	Missing	31 (0.1) [0.1]	NS	1.39 (0.45 to 4.28)	NS	1.7 (0.61 to 4.76)
Was antenatal care available?	No	3695 (15.6) [16.7]		Not in multivariate model		Baseline
	Yes	13 186 (55.8) [55.4]		—	0.001	1.46 (1.16 to 1.83)
	Missing	6743 (28.5) [27.9]		—	NS	0.38 (0.12 to 1.21)
Place of child’s birth	Home birth	8729 (36.9) [42.5]		Not in multivariate model		Baseline
	Government hospital	2594 (11.0) [10.8]		—	<0.001	1.57 (1.22 to 2.02)
	Private hospital	5526 (23.4) [15.8]		—	NS	1.07 (0.87 to 1.32)
	Missing	6775 (28.7) [30.9]		—	NS	2.62 (0.82 to 8.35)
Mothers education	Primary	4007 (17) [16.1]		Baseline		Not in multivariate model
	High	4926 (20.9) [18.6]	0.001	1.57 (1.19 to 2.07)		—
	Middle	2028 (8.6) [7.6]	0.02	1.37 (1.05 to 1.81)		—
	Missing	12 663 (53.6) [57.6]	<0.001	0.65 (0.54 to 0.77)		—
Access to a radio	No	13 934 (59) [44.1]		Baseline		Not in multivariate model
	Yes	9673 (40.9) [55.9]	0.028	0.75 (0.58 to 0.97)		—
	Missing	18 (0.1) [0.1]	NS	0.58 (0.09 to 3.8)		—
Access to a television	No	9544 (40.4) [91.1]		Baseline		Baseline
	Yes	14 062 (59.5) [8.8]	0.003	1.28 (1.09 to 1.5)	0.047	1.26 (1 to 1.58)
	Missing	18 (0.1) [0.1]	NS	1.1 (0.05 to 23.73)	NS	1.52 (0.32 to 7.24)
Wealth Index	Linear on calculated wealth quintiles	NA	0.004	1.12 (1.04 to 1.21)	0.05	1.1 (1 to 1.2)
Province	Punjab	21 880 (92.6) [91.8]		Baseline		Baseline
	Balochistan	1744 (7.4) [8.2]	<0.001	0.19 (0.15 to 0.24)	<0.001	0.3 (0.24 to 0.37)
Age	0	5964 (25.2) [26]		Baseline		Baseline
	1	8006 (33.9) [33.8]	<0.001	4.22 (3.57 to 5)	<0.001	8.74 (6.95 to 11.01)
	2	9654 (40.9) [40.2]	<0.001	6.38 (5.37 to 7.57)	<0.001	16.28 (12.22 to 21.68)

NS: not significant.

### Comparison of routine immunization coverage from AFP and MICS data

**Figure 3.**
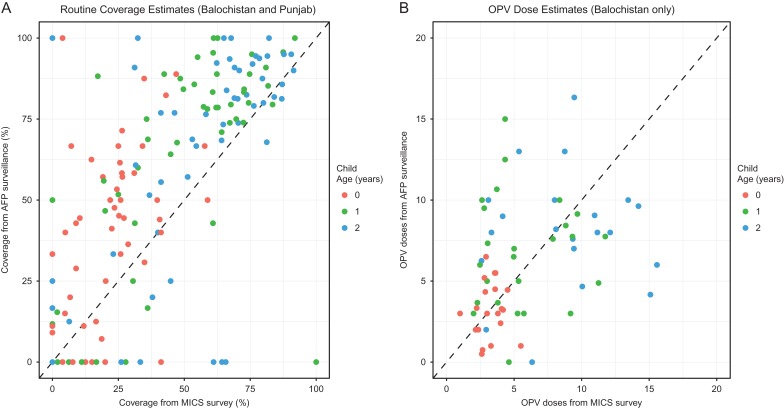
Comparison of district age estimates of (A) pentavalent coverage administered via routine immunization and (B) total OPV doses, reported via MICS and AFP surveillance. The diagonal line indicates complete agreement between data.

There were 2649 non-polio AFP cases from districts included in the Balochistan and Punjab MICS that met the inclusion criteria. There was a strong positive correlation between MICS- and AFP-based estimates of routine pentavalent coverage (r=0.649 [95% CI 0.55 to 0.73], p<0.01; Figure 3A). AFP-based estimates were 28.2% (80% CI −2.8 to 88.4) higher than MICS estimates. This was consistent across all ages and was significantly (p<0.01) higher within Punjab. Total OPV dose estimates (analysed only in Balochistan) had moderate agreement between data sources, illustrating a positive but weaker correlation (r=0.348 [95% CI 0.12 to 0.54], p<0.01). AFP-based estimates were lower, with a −3.5% (80% CI −68.2 to 160.2) reduction when compared with MICS estimates, with more variation between observations that increased with age and OPV dose (Figure 3B).

## Discussion

This study has identified considerable variation in routine immunization rates across two provinces in Pakistan, suggesting large inequalities in healthcare. Compared with previous estimates of coverage from DHS and other national surveys, such as the Pakistan Social and Living Standards Survey (PSLSS), the disparity between provinces is consistent. Pentavalent vaccination reported here is lower in both provinces when compared with other data sources: the PSLSS reported pentavalent coverage of 57% and 94% for Balochistan and Punjab, respectively, in 2012 compared with the 20.7% and 59.7%, respectively, reported here.^16^ A DHS in 2012 reported pentavalent coverage of 27.1% and 76.3% in Balochistan and Punjab,^17^ respectively, which is more consistent with the findings reported here (Table 2). OPV coverage is high in both provinces and it is likely that frequent supplementary immunization campaigns have made a significant contribution to this. MV coverage reported here is lower than that reported in the PSLSS (55% and 89%, respectively) and similar to DHS estimates (37.3% and 70.0%, respectively). MV coverage is higher than pentavalent coverage, which likely reflects an increased use of measles supplementary immunization, whereas the pentavalent vaccine is mostly administered via health centres. The national estimates are informed by survey data and government administrative data, and are adjusted based on the perceived data quality and other factors within the WHO/UNICEF methods described by Burton et al.^18^ National estimates are typically higher than the estimates from Balochistan, as coverage from the remaining provinces is similar to estimates within Punjab. The national pentavalent estimate was largely based on the national estimates from the 2011 DHS (65.2%), with some adjustment.

**Table 2.** Comparison of vaccination coverage of the pentavalent, OPV and MV for Balochistan and Punjab according to different sources

Vaccine	Province	Source	Adjusted % vaccinated
Pentavalent	Country	WUENIC	74
	Balochistan	PSLSS	57
		DHS	27.1
		MICS	20.7
	Punjab	PSLSS	94
		DHS	76.3
		MICS	59.7
OPV	Country	WUENIC	75
	Balochistan	PSLSS	91
		DHS	74.9
		MICS	80.7
	Punjab	PSLSS	96
		DHS	95.2
		MICS	88.8
Measles	Country	WUENIC	53
	Balochistan	PSLSS	55
		DHS	37.3
		MICS	39.7
	Punjab	PSLSS	89
		DHS	70
		MICS	74

DHS: Demographic Health Survey (2012); MICS: Multiple Indicator Custer Survey (2011); PSLSS: Pakistan Social and Living Standards Survey (2011); WUENIC: WHO/UNICEF Estimates of National Coverage (2011).

Comparison of AFP and MICS data illustrates considerable consistency in district age observations, and this finding is reassuring. However, AFP estimates were consistently higher than MICS estimates within a district (and significantly so in Punjab), suggesting that there might be increased access to vaccination in AFP-affected children or an increased bias on recall of vaccination histories from cases of AFP when compared with cross-sectional data. AFP-based estimates of pentavalent coverage are inferred from OPV doses received via routine immunization, so this finding is reliant on assuming that there are minimal stock-outs of the pentavalent vaccine when receiving the OPV. Recall-based estimates of coverage have not been previously compared and this finding is important considering that these data are used to inform health metrics such as WHO/UNICEF estimates and vaccination planning activities. Analysis of additional datasets from other countries may help identify the source of this inconsistency. We identified more variation in estimates in the total OPV doses reported than in pentavalent coverage. The OPV is administered via routine and supplementary vaccination activities, where supplementary vaccination coverage is known to vary within spatial units smaller than the district level.^19,20^ As the total number of OPV doses received increases, the error in recall is also likely to increase, and this may explain the reduced correlation in total OPV doses.

The education of the mother has been consistently associated with routine vaccination across many settings. Maternal education captures important aspects of social exclusion in Pakistan; for example, only 14.3% of mothers had received higher education. Improving educational attainment is likely to be an onerous task and, in the interim, strategies that focus on improving health-seeking behaviours of parents with little education should be a priority within Pakistan. Along a similar thread, the association of use of antenatal care and children being born within government hospitals may be related to increased health literacy and exposure to healthcare messaging, which includes promotion of vaccination. The association of access to a television and vaccination also points towards increased health awareness, which may be facilitated through messages made through this medium, although other communication sources are thought to be more effective.^21^ Additionally, access to a radio was associated with reduced pentavalent coverage. While the significance of this association was moderately strong (p=0.028) and not significant for measles, this finding may be considered counterintuitive. It is unclear whether this association is a type I error, possibly due to confounding effects. As the analysis is included within a multivariate regression analysis that includes wealth, identification of these risk factors is independent of their association with wealth.

To facilitate improvements in routine vaccination, the National Emergency Action Plan for Polio Eradication has prioritized joint work plans between polio eradication and provincial EPI personnel.^22^ In addition, the government of Pakistan recently developed a comprehensive multiyear plan for improving routine immunization that includes target setting of measles morbidity reduction, interruption of polio transmission and elimination of neonatal tetanus by 2018.^23^ This ambitious plan includes the monitoring of EPI vaccine coverage, increased human resource management, increased funding, increased investment in cold-chain logistics, improvements in service delivery, communication and advocacy planning and province-specific implementation plans. It is believed that the devolution of health to provinces offers a unique opportunity to focus attention on the health and nutrition of the poor, and with it a focus on improving routine immunization.^24^ The key to success will be effective implementation of these strategies and in making long-term investments to address the social determinants of health inequalities.

The analysis presented here is subject to several limitations. We have explored household factors that were associated with vaccination coverage, but healthcare access and service provision will also influence vaccination. We were unable to incorporate data that describe these factors explicitly. A comprehensive analysis of routine vaccination services within Pakistan by Masud and Navartne^3^ suggested that stagnation in provision of EPI services was associated with weak capacity building, poor local planning, limited financial resources and reduced monitoring and evaluation of services. While we identified a strong correlation between AFP and MICS data sources, more reliable information may be gained from the regular use of vaccination cards by families and recording this information within AFP surveillance. Within the AFP data, we assumed that OPV doses received via routine immunization were equivalent to pentavalent doses, enabling comparisons to MICS data. This assumption relies on there being a good availability of the pentavalent vaccine within health centres in Pakistan. Should stock-outs be common in Pakistan, this assumption will no longer be valid. There were also issues with data quality within the MICS. More than 50% of observations were excluded from the analysis because vaccination history was not recorded and explanatory variables (such as education, child’s place of birth and use of antenatal care) had many missing responses, which may increase type II errors in the analysis and may make the survey prone to biases in responses. Only missing data on the mothers’ education were associated with vaccination coverage, suggesting that this finding should be treated with caution. Additionally, the sample size calculations and population stratification of the survey were based on data from the 1998 census, which is likely to be outdated. A recent (2017) census in Pakistan will likely alleviate issues associated with inaccurate demographic data.^25^ Cross-sectional surveys only provide indicators of association and these may not always provide an obvious course of action to improve vaccination coverage. A recent systematic review identified (with moderate certainty) that providing information and discussing vaccination with parents at village meetings or at home probably improves coverage.^26^ The authors also reported that there was limited evidence of effective interventions and consequently there is a substantial need to carry out well-conducted trials of interventions to improve routine immunization coverage within settings such as Pakistan.

## Conclusions

Routine vaccination within Pakistan exhibits much regional variation that is associated with factors relating to gender equity and access to education. Although the analysis supports an increasing body of evidence that vaccination rates have stagnated in Pakistan in recent years, considerable efforts have been put in place to address these observations.
